# Ulnolunate Impaction Syndrome

**Published:** 2015-01-08

**Authors:** Saptarshi Biswas

**Affiliations:** Westchester University Medical Center, Valhalla, NY

**Keywords:** ulnar impaction, ulnar impaction syndrome, ulnar wrist pain, positive ulnar variance, subchondral sclerosis

**Figure:**
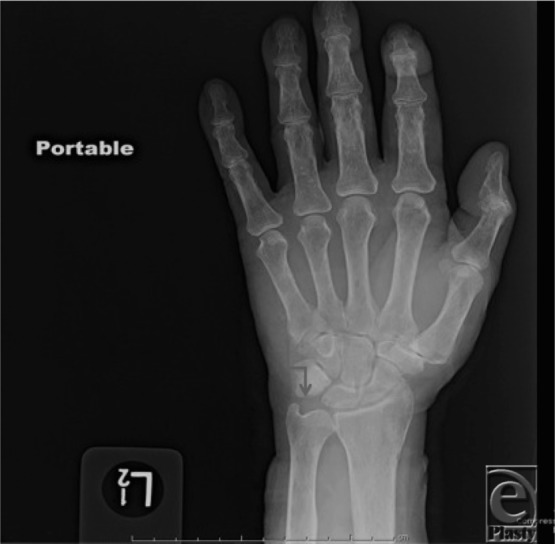


**Figure:**
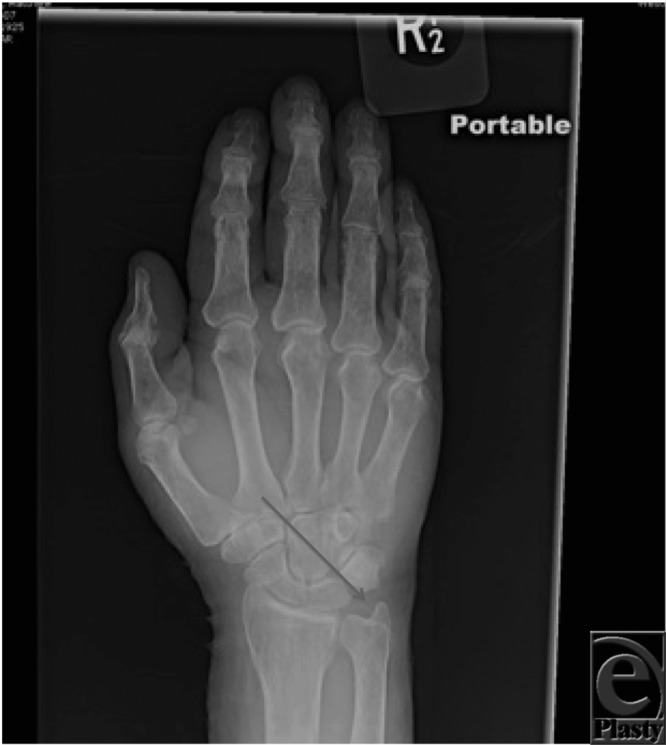


## DESCRIPTION

A 65-year-old patient presented with persistent ulnar-sided pain for years. X-ray revealed no osseous or articular abnormality but mild to moderate basal joint degenerative arthrosis. There was positive ulnar variance bilaterally with cystic changes along the ulnar aspect of the lunate left greater than right, suggestive of “ulnolunate impaction syndrome.”

## QUESTIONS

1. **What is the diagnosis?**
2. **Is there any eponym?**
3. **What are the etiology and clinical presentation of the condition?**
4. **What are the treatment options for ulnar impaction syndrome (UIS)?**

## DISCUSSION

Ulnar impaction syndrome is a degenerative wrist condition caused by the ulnar head impacting upon the ulnar-sided carpal bones. This abutment results in increased load bearing across the ulnar head, triangular fibrocartilage complex (TFCC), and ulnar carpals and subsequent degeneration of the TFCC, chondromalacia of the osseous structures involved (ulna and carpals, especially the lunate), and disruption of the triquetrolunate ligament.^1^

This condition is also known as ulnar abutment or ulnocarpal loading.

The most common predisposing factor is positive ulnar variance, an increased ulnar length relative to the radius. In the positive ulnar variance wrist, the TFCC is stretched and thin, and greater biomechanical forces, specially rotation forces, impact the joint. This positive variance can be congenital^1^ or acquired^2^ radial shortening secondary to trauma—for example, a malunion of the radius after a distal radius fracture, an Essex-Lopresti injury, proximal migration of the radius after radial head excision, or premature physeal closure of the radius.^1^^-^3 Wrists without positive ulnar variance or “ulnar neutral” or “ulnar negative” can also acquire UIS because variance can increase during functional activities, especially forearm pronation and gripping.^3^^,^^4^ When ulnar variance increases in wrists that are ulnar negative or neutral (and thus have a thicker TFCC), ulnocarpal load also increases.^5^ Therefore, although UIS is most common in those with an ulnar positive wrist, it can also occur in wrists with either negative or neutral variance.^4^ Ulnar impaction syndrome is insidious and progressive, so patients can be asymptomatic and have the syndrome or severely symptomatic. Pain, occasional edema, decreased range of motion of the wrist, decreased forearm rotation, and tenderness to palpation dorsally just distal to the ulnar head and just volar to the ulnar styloid process are the common complaints. Forceful grip, forearm pronation, and ulnar deviation aggravates.^5^

Conservative methods should be attempted as the first line of treatment and include immobilization for 6 to 12 weeks, nonsteroidal anti-inflammatory drugs, corticosteroid injection and limiting aggravating movements such as pronation, gripping, and ulnar deviation.^5^ The operative options consists of (A) Ulnar shortening osteotomy,^2^^,^^6^ where the ulna is shortened, with removal of 2 to 3 mm of shaft and fixated with a compression plate (tubular or standard); (B) Arthroscopic wafer procedure,^7^ where 2 to 4 mm of cartilage and bone removed from under TFCC arthroscopically and indicated if no lunotriquetral instability with minimal ulnar variance, cystic changes of carpus on radiographs, and evidence of degeneration of TFCC on magnetic resonance imaging; (C) Bowers procedure,^8^ which involves resection of ulnar articular head, leaving shaft and styloid relationship intact; (D) Darrach procedure,^8^ where the ulnar head is excised if TFCC is not able to be reconstructed; (E) Sauve-Kapandji procedure,^8^ where resection of distal ulna and fusion of ulnar head and radius via screw and/or pins is done. This is a good option for manual laborers; and (F) Ulnar head replacement specially for severe ulnocarpal arthrosis and as a salvage for failed Darrach.

Several studies have reported a high percentage of success with ulnar shortening osteotomy. Baek et al^2^ showed significant improvements for idiopathic UIS, where the postoperative modified Gartland and Werley scores (most commonly described instrument in literature for evaluating outcome after wrist surgery) improved significantly from the preoperative score. There was also reduced subluxation of distal radioulnar joint, resolve of degenerative cystic changes of ulnar carpal bones, as well as reduction in average ulnar variance from +4.6 preoperatively to −0.07 postoperatively. Chun et al^6^ showed 100% union in 6 to 8 weeks, 72% excellent results on Gartland and Werley score. In arthroscopic wafer procedure,^7^ 85% to 100% of patients showed good to excellent results with nearly full range of motion. However, grip strength did not improve and patients with a history of distal radius fracture had increased pain after surgery. Feldon et al reported 69% excellent and 31% good results for open wafer procedure although it required longer postoperative immobilization and recovery. Tomanino et al,^4^ using combined arthroscopic TFCC debridement and wafer resection, reported total pain relief in 67% patients along with 36% increase in grip strength.
